# Supplementary figures and images for: Comparison of the composition and function of gut microbes between adult and juvenile Cipangopaludina chinensis in the rice snail system
Source: PeerJ. 2022 Mar 8;10:e13042. doi: 10.7717/peerj.13042 (PMC8916024; doi:10.7717/peerj.13042)

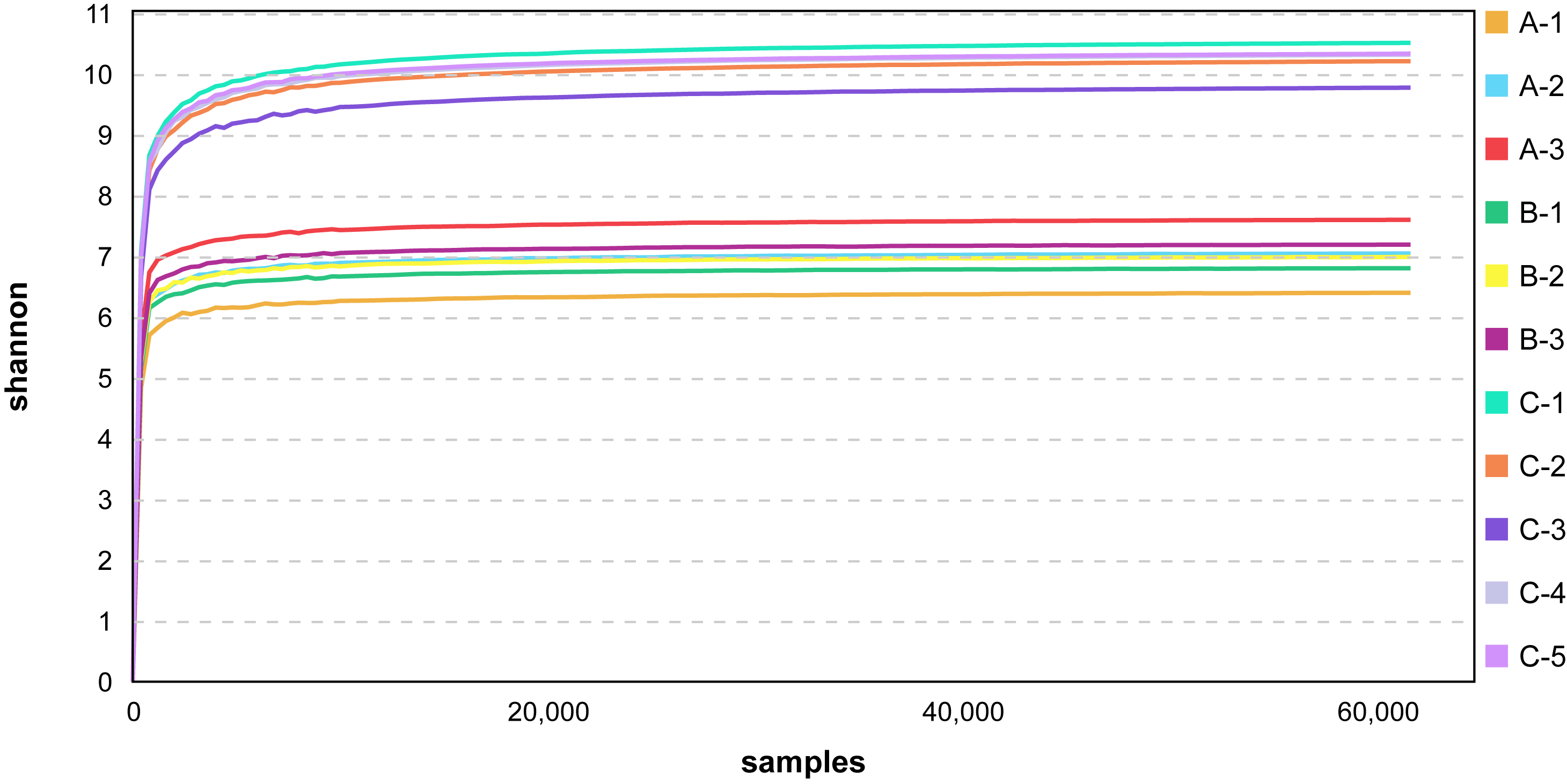

Supplement: Supplemental Information 1 — A1–A3: adult snails gut samples. B1–B3: juvenile snails gut samples. C1–C5: sediment samples. [file peerj-10-13042-s001.png]
